# Algorithms for automated detection of hook effect-bearing amplification curves

**DOI:** 10.1016/j.bdq.2018.08.001

**Published:** 2018-10-16

**Authors:** Michał Burdukiewicz, Andrej-Nikolai Spiess, Konstantin A. Blagodatskikh, Werner Lehmann, Peter Schierack, Stefan Rödiger

**Affiliations:** a Technical University of Warsow, Warsow, Poland; b University Medical Center Hamburg-Eppendorf, Hamburg, Germany; c Pirogov Russian National Research Medical University, Moscow, Russia; d Attomol GmbH, Lipten, Germany; e Institute of Biotechnology, Brandenburg University of Technology Cottbus – Senftenberg, Senftenberg, Germany

**Keywords:** Hook effect, QPCR, TaqMan, EvaGreen, SybrGreen, Linear, Nonlinear, RDML, Automation

## Abstract

Amplification curves from quantitative Real-Time PCR experiments typically exhibit a sigmoidal shape. They can roughly be divided into a ground or baseline phase, an exponential amplification phase, a linear phase and finally a plateau phase, where in the latter, the PCR product concentration no longer increases. Nevertheless, in some cases the plateau phase displays a negative trend, e.g. in hydrolysis probe assays. This cycle-to-cycle fluorescence decrease is commonly referred to in the literature as the *hook effect*. Other detection chemistries also exhibit this negative trend, however the underlying molecular mechanisms are different.

In this study we present two approaches to automatically detect hook effect-like curvatures based on linear (*hookreg*) and nonlinear regression (*hookregNL*). As the hook effect is typical for qPCR data, both algorithms can be employed for the automated identification of regular structured qPCR curves. Therefore, our algorithms streamline quality control, but can also be used for assay optimization or machine learning.

## 1 Introduction

Key elements of any PCR assay are the primers since they control the sensitivity and specificity of the reaction [1]. Not less important is a stable binding of probes to the amplicon for the generation of a meaningful amplification curve signal in quantitative real-time PCR (qPCR). For hybridization probes, a phenomenon observed in late cycles is the competition between amplicon strands and the probes, which may reduce the fluorescent signal considerably [2]. This so-called *hook effect* is often observed at high template concentrations that are typical in later cycles [3], and where the single strands of the amplicons re-anneal faster than the probes with the amplicons. For detection chemistries other than hybridization probes, such as hydrolysis probes, hook effects have not been described. Other reasons such as the nuclease activity of the polymerase may also contribute to the decreasing probe signal [4], however it is more likely that this results in an earlier plateau phase.

Several experimental conditions can be adjusted to minimize the hook effect in qPCR, such as optimizing the DNA template, probe or MgCl_2_ concentrations, reducing the cycle number or conducting asymmetric PCR [3]. Although the decrease in fluorescence does not affect the efficiency or specificity of amplification and target detection [3], a low fluorescence signal can entail limitations to the sensitivity of the assay, including melting curve and data analysis. For example, it was reported that the hook effect is challenging genotyping and forensic applications [2], [5], [6].

**Fig. 1:**
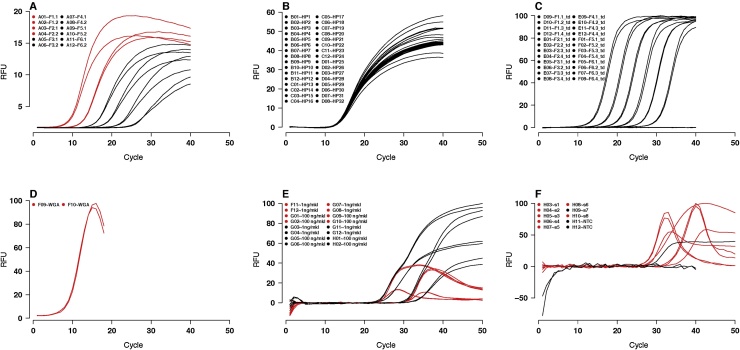
Overview of the data included in *hookreg.rdml*. The amplification curves exhibit different curvatures, including sigmoidal shapes, curves with hook effect-like shapes and negative qPCR reactions. For fluorescence readout, EvaGreen, SYTO-13 and TaqMan probes were employed. Black amplification curves display no hook effect, while red amplification curves do, as identified by our analysis pipeline. (A) A01-A12: SYTO-13, (B) B01-D08: hydrolysis probes, (C) D09-F08: SybrGreen I, (D) F09-F10: EvaGreen, (E) F11-H02: hydrolysis probes, (F) H03-H12: hydrolysis probes. Single plots for all amplification experiments are shown in Supplemental Fig. 1. RFU, relative fluorescence units.

Although the hook effect has been reported mainly for HybProbes and other specific detection chemistries [4], [7], interestingly, it can also be encountered in amplification reactions with intercalating dye chemistries (Fig. 1A, D) as well as hydrolysis probes (TaqMan™; Fig. 1E, F). For the latter, hook effects are evident in the non-baselined raw data (data not shown). Here, the mechanistic basis for decreasing fluorescence in late cycles are hitherto unknown, however photobleaching or requenching may pose possibilities.

On the software side, Nolan et al. [8] clearly demonstrated that a wrong baseline setting can lead to up- or downward sloping baseline estimates, resulting in the plateaus sloping to the opposite direction. Here, it should be noted that most qPCR systems apply a fitted trendline to a number of early cycles in the fluorescence baseline.

Algorithms to detect hook effect-like curve structures in the tail region can be employed for quality control. For example, a deviation from a sigmoidal model can be indicative for a failed experiment, and these amplification curves should undergo a more optimized analysis procedure or even be excluded from further analysis. A plethora of software packages for the analysis of qPCR data have been published [9], but none of these appear to describe algorithms that can be used to detect hook effects. This study revised existing software and demonstrates two approaches to detect hook effects in qPCR curvatures. One algorithm is based on maximum peak finding and linear regression (*hookreg*), while the other is based on fitting a non-linear six-parameter model to acquire a coefficient for the slope of the plateau phase (*hookregNL*).

## 2 Materials and methods

The following algorithmic steps describe both the *hookreg()* and *hookregNL()* methods that are applied to raw fluorescence data without baseline correction. If done correctly, these are also applicable to baseline-corrected values. The input values *x*_*i*_ and *y*_*i*_ stand for the cycles and cycle dependent fluorescence intensity, respectively. The return values are the slope estimate and its *p*-value, the slope's confidence interval and a logical decision if a hook-effect is present. *hookregNL()* uses core functionality from the qpcR package [10], [11]. Both algorithms are implemented in the PCRedux package v. 0.2.6-1 (https://cran.r-project.org/package=PCRedux) of the R statistical computing language (https://www.r-project.org).

***hookreg()*:**

• Find *x*_0_ = cycle at max(*y*_*i*_).
• Fit a linear model *y*_*i*_ = *β*_0_ + *β*_1_*x*_*i*_ + *ε* from *x*_0_ to *x*_*n*_, where *n* = length of *x* (number of cycles), when at least five cycles can be employed.
• Calculate the *p*-value for estimated slope ${\hat{\beta }}_{1}$ as well as its 99.75% confidence interval CI.
• If *p* < 0.0025 (one-sided t-test), then a hook-effect is defined as being present, because the slope of ${\hat{\beta }}_{1}$ is significantly different from 0 (no slope) and negative. If both CI_0.125%_ and CI_99.875%_ bounds are negative, we can be 99.75% certain that 0 (no slope) is not included in the CI of ${\hat{\beta }}_{1}$.

**Fig. 2:**
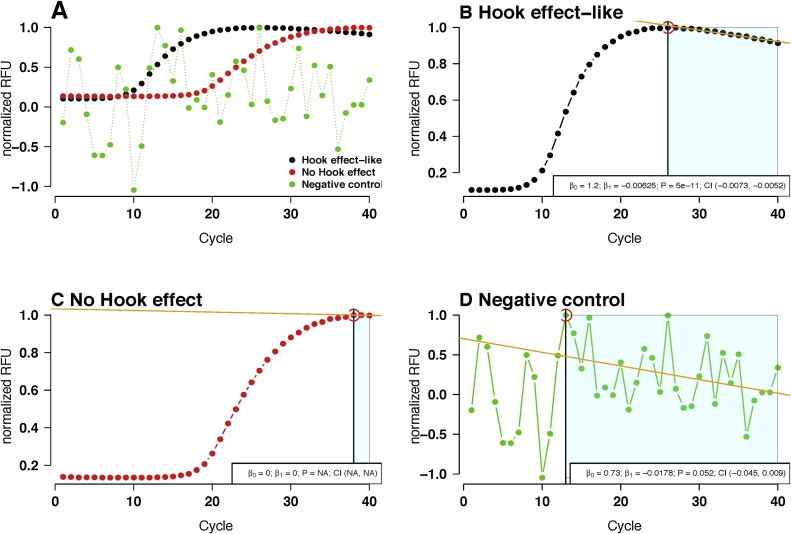
Hook effect analysis of amplification curves from different samples. The amplification plots are shown without background subtraction. The samples were taken from the *hookreg.rdml* data set (see Supplemental Files). (A) Overview of the amplification curves. Black: Amplification curve with a hook effect-like curvature. Red: Amplification curve with a standard plateau. Green: Negative amplification reaction. (B) All values from *x*_0_ to *x*_*n*_ in the ROI (green) are used for ordinary linear regression. Presence of a hook effect was tested by the slope of the fit (*p* < 0.005) or a negative 99.5% confidence interval. (C) Amplification curves with no hook effect, no meaningful regression results from the lack of at least 5 cycles after *x*_0_. (D) Negative amplification curves get discarded by a logical decision. RFU, relative fluorescence units; normalized RFU, relative fluorescence units normalized to the 99th percentile. *β*_0_, intercept; *β*_1_, slope; P, p-value; CI, confidence interval; NA, missing value.

A visual representation of the algorithm is shown in Fig. 2.

***hookregNL()*:**

• Remove the first 5 (or more) cycles *x*_1_, *x*_2_ … *x*_5_ from the data to minimize slope effects in the baseline region.
• Fit a six-parameter log-logistic model *y*_*i*_ = *c* + *kx*_*i*_ + (*d* − *c*)/(1 + exp(*b*(log(*x*_*i*_) − log(*e*))))^*f*^ by nonlinear least-squares. This model has the following parameters: *c*: lower asymptote (*baseline*); *d*: upper asymptote (*plateau*); *k*: linear slope; *b*: sigmoidal slope; *e*: point-of-inflection (if *f* = 1); *f*: asymmetry around point-of-inflection.
• Calculate the 99.75% confidence interval CI for the estimated slope parameter $\hat{k}$.
• If both CI_0.125%_ and CI_99.875%_ bounds are negative, then a hook-effect is defined as being present, because a slope of 0 (no slope) is not included in CI.

Confidence intervals instead of slope estimates for both functions are employed to deliver significance to the plateau phase's fluorescence decrease.

For algorithm evaluation, we compiled a data set consisting of sigmoidal qPCR curves with no hook effect, non-sigmoidal curves (negative control), curves with no plateau phase, curves with a slight negative trend (hook effect-like) and curves with pronounced hook effect (see also Supplement Fig. 1, Supplement Table 1). The raw data were compiled from the *boggy* data set [12], the *testdat* data set [11], the *C127EGHP* data set [13], a whole genome amplification experiment [14] and from an in-house *BRCA1* gene quantification experiment. The raw data were rated by the *humanrater()* function either as hook effect-bearing (“y”) or not (“n”), as described in the Supplementary Information of [13]. As reproducible research is an important aspect in science [15], [16], the amplification curve data (Fig. 1) were combined and made available as an RDML file [17] that was used for all analyses.

## 3 Results and discussion

In general, the presence of hook effects should be checked during the analysis of qPCR experiments. For example, when fitting models that utilize all data points, the *Cq* value is highly influenced by the magnitude of the plateau phase [18], which will be estimated in a four-parameter sigmoidal model approximately as the mean of all plateau phase values. Hence, the stronger the hook effect, the lower the plateau's mean. In contrast, methods that do not include the hook region fluorescence values and fit only the exponential region [19], [20], [21] or are parameter-free (e.g. splines), will not be influenced by the presence of a downward-sloped plateau. Using both algorithms in combination for quality control is feasible for the identification of regular structured qPCR curves prior to *Cq* value and qPCR efficiency estimation. In the presence of a hook-like plateau, the user is encouraged to check that (i) the baseline setting is correct, (ii) the assay is optimized and (iii) a quantitation algorithm independent of plateau phase values is employed.

In this work, we introduce the *hookreg()* and *hookregNL()* algorithms to identify hook effect-bearing qPCR amplification curves. The first, *hookreg()*, is based on standard linear regression and can be easily implemented in less sophisticated statistical software packages or spread-sheet applications. The second, *hookregNL()*, is based on the fitting of a six-parameter sigmoidal model, available in statistical software packages such as qpcR for the R statistical computing language [10], [11], but also definable in the “Nonlinear Regression” menu of many softwares.

Both algorithms are implemented in the PCRedux package, and their output provides the fit parameters as well as corresponding *p*-values, the cycle when the hook presumably starts (*hookreg()*), the confidence intervals, and finally the decision about the presence or absence of a hook effect (Supplement Section 4). *hookreg()* makes some assumptions about the data, e.g. at least five additional data points (5 cycles) from the maximum are required for a linear fit, so that curves with less points will be ignored. This may lead to false negative results. The *hookregNL()* algorithm makes the assumption that a sigmoid model can be fitted. A few cycles (by default 5, but extendable) of the baseline region need to be removed prior to fitting to avoid false positives from a downward sloping baseline (Fig. 1). Both algorithms were compared and gauged against a human classification of the *hookreg.rdml* data set and displayed different performances, resulting in false negative and false positive decisions in some cases (Supplement Table 2 and Supplement Table 3).

Interestingly, both algorithms complemented each other and complete agreement was observed (Table 1) when they were combined by a logical statement (see Supplement Section 5). Essentially, both algorithms aim to detect negative trends (hook effect or hook effect-like curvatures) in the tail region. In the *hookreg.rdml* data set we achieved 100% sensitivity and 97% specificity with the combined approach. In addition, we observed cases where the negative trend was not immediately evident to the human rater, but the statistical estimates obtained from *hookreg()* and *hookregNL()* indicated a significant downward slope. Since both the *hookreg()* and *hookregNL()* functions report the steepness of the slope (Supplement Tables 2 and 3), users can decide if the data have a hook effect-like curvature. Corroborating their high performance, we observed no false negative classification by both algorithms within the *hookreg.rdml* data set.

**Table 1:** Analysis of the performance of both algorithms. The performance measures were determined with the *performance()* function of the PCRedux package. See Supplement Section 5.

	hookreg	hookregNL	combined
Sensitivity	0.90	0.33	1.00
Specificity	0.97	1.00	0.97
False positive rate	0.03	0.00	0.03
False negative rate	0.10	0.67	0.00
Accuracy	0.96	0.85	0.98
True positive	19	7	21
True negative	73	75	73
False positive	2	0	2
False negative	2	14	0

Finally, a graphical user interface for both algorithms was introduced to the *rdmlEdit()* function of the RDML R package [17], which is available as a web server or can be deployed locally (see Supplement Section 6). This graphical user interface enables testing qPCR curves for hook effects and marks these, depending on the result.

## Supplemental files

**Installation**: The installation of the PCRedux package is described in the Supplement Section 2 and at https://github.com/devSJR/PCRedux. The functions can be used in software such as RKWard [22] in combination with the RDML package (≥ v. 0.9-9) by invoking the *rdmlEdit()* function (for details see [17]).
**hookreg.rdml**: The RDML file containing the amplification curve data. The file can also be accessed via https://github.com/devSJR/PCRedux/blob/master/inst/hookreg.rdml.
**SI1.pdf**: Supplement with the results of the data analysis.
http://shtest.evrogen.net/rdmlEdit/ link to the rdmlEdit graphical user interface web server

## Funding

This work was funded by the Federal Ministry of Education and Research (BMBF) InnoProfile-Transfer-Project 03IPT611X and in part by the Gesundheitscampus Brandenburg “digilog: Digitale und analoge Begleiter für eine alternde Bevölkerung”, Brandenburg Ministry for Science, Research and Culture (MWFK).

## Conflicts of interest

Werner Lehmann is a shareholder and employee of Attomol GmbH. The other authors have no conflicts of interest to declare.
