# Supplementary material for: Algorithms for automated detection of hook effect-bearing amplification curves
Source: Biomol Detect Quantif. 2018 Oct 16;16:1–4. doi: 10.1016/j.bdq.2018.08.001 (PMC6287529; doi:10.1016/j.bdq.2018.08.001)
Supplement: Supplementary file 1 [file mmc1.pdf]

Supplement for “Algorithms for Automatized Detection of Hook Effect-bearing Amplification Curves”

Michał Burdukiewicz[a], Andrej-Nikolai Spiess[b], Konstantin A. Blagodatskikh[c], Werner Lehmann[d], Peter Schierack[e], and Stefan Rödiger[e]

[a]Technical University of Warsow, Warsow, Poland; [b]University Medical Center Hamburg-Eppendorf, Hamburg, Germany; [c]Pirogov Russian National Research Medical University, Moscow, Russia; [d]Attomol GmbH, Lipten, Germany; [e]Institute of Biotechnology, Brandenburg University of Technology Cottbus – Senftenberg, Senftenberg, Germany

This version was compiled on September 9, 2018

This is a supplemental document for the study *Algorithms for Automatized Detection of Hook Effect-bearing Amplification Curves*. Quantitative real-time PCR (qPCR) is a widely used method for gene expression analysis, forensics and medical diagnostics (Dvinge and Bertone, 2009; Martins *et al.*, 2015; Sauer *et al.*, 2016).
Numerous algorithms have been developed to extract features from amplification curves such as the cylce of quantification and the amplification efficiency (Ruijter *et al.*, 2013). There is an agreement, that these algorithms need to be evaluated and benchmarked for their performance (Kemperman and McCall, 2017). But at an earlier level it is important to have a solid foundation for the data preprocessing (Spiess *et al.*, 2015, 2016; Ronde *et al.*, 2017). Digitalization of processes holds the promise that potential human mistakes can be spotted and that diagnostic processes can be automatized. The aim of the study is to provide software tools and algorithms, which assist qPCR users during the analysis of their data. In particular, this study shows how it is possible to automatically detect hook effects (see Barratt and Mackay (2002)) or hook effect-like curvatures.

hook effect | RDML | qPCR | automatized | machine learning

1. Introduction

The functions and data presented in the paper are available from https://github.com/devSJR/PCRedux. The data, including the RDML file, are part of the `PCRedux` package and are made available in the CSV or RDML format (Rödiger *et al.*, 2017) for vendor independent analysis.

All analyses were implemented and conducted with the **R** statistical computing language (R Core Team, 2017; Rödiger *et al.*, 2015a) and dedicated integrated development environments such as **RKWard** (Rödiger *et al.*, 2012). Further documentation can be found in the help files of the **R** packages.

2. Installation

The **hookreg()** and **hookregNL()** functions are part of the `PCRedux` package for the **R** statistical computing language. Download from CRAN http://cran.r-project.org/ the **R** version for the required operating system and install **R**. Then start **R** and type in the prompt:

```
# Select your local mirror
install.packages("PCRedux")
```

The `PCRedux` package should just install. If this fails make sure you have write access to the destination directory and follow the instructions of the **R** documentation:

```
# The following command points to the help for download and install of packages
# from CRAN-like repositories or from local files.
?install.packages()
```

The package can be installed as the latest development version using the devtools R package.

```
# Install devtools, if you haven't already.
install.packages("devtools")

library(devtools)
install_github("devSJR/PCRedux")
```

It is recommended to use software with an integrated development environment such as `RKWard` Rödiger *et al.* (2012). To work with RDML data it is recommend to use the *RDML* package ($\geq_{v.}$0.9-9) by invoking the **rdmlEdit()** function (for details see Rödiger *et al.* (2017)) or the rdmlEdit GUI web server (section 6). The RDML file `hookreg.rdml` contains the amplification curve data. However, other software package (e.g., (Lefever *et al.*, 2009; Ruijter *et al.*, 2015)) can also be used to work with the RMDL data file format.

3. Results for the analysis of the `hookreg.rdml` data set by humanrater()

All calculations in the following sections were employed on the `hookreg.rdml` data set, which is part of the `PCRedux` package. The data were transfered to the **R** environment by the `RDML` package (Rödiger *et al.*, 2017). An overview of the used samples and the qPCR detection chemistries and the classification by two humans ("Hook effect-like Rater 1", "Hook effect-like Rater 2") is shown in Table 1.

Loading experiment: exp1 run: run1 All amplification curves were plotted according to their experiment conditions. They differed in the target molecules (e.g., *MLC-2v*, *BRCA1*) and the detection chemistries (e.g., EvaGreen, SybrGreen, hydrolysis probes). Figure 1 shows seven plots for the corresponding experiments. The amplification curves were not preprocessed to preserve the curvature. Selected amplification curves were noisy (e.g., Figure 1F), had overshots or undershot in the background phase (e.g., Figure 1E-G), a short hook phase (e.g., Figure 1D). Amplifcation curves of Figure 1A, D, F and F exhibited a clearly visible hook effect or a hook like effect.

```r
par(mfrow=c(4,2))

# Plot all data of the hookreg.rdml-file according to their type.
# Synthetic template, detected with Syto-13
matplot(data[, 1], data[, 2:13], type="l", lty=1, lwd=2, ylab="RFU", xlab="Cycle")
mtext("A", cex = 1.8, side = 3, adj = 0, font = 2)

# Human MLC-2v, detected with a hydrolysis probe.
matplot(data[, 1], data[, 14:45], type="l", lty=1, lwd=2, ylab="RFU", xlab="Cycle")
mtext("B", cex = 1.8, side = 3, adj = 0, font = 2)

# S27a housekeeping gene, detected with SybrGreen I.
matplot(data[, 1], data[, 46:69], type="l", lty=1, lwd=2, ylab="RFU", xlab="Cycle")
mtext("C", cex = 1.8, side = 3, adj = 0, font = 2)

# Whole genome amplification, detected with EvaGreen.
matplot(data[, 1], data[, 70:71], type="l", lty=1, lwd=2, ylab="RFU", xlab="Cycle")
mtext("D", cex = 1.8, side = 3, adj = 0, font = 2)

# Human BRCA1 gene, detected with a hydrolysis probe.
matplot(data[, 1], data[, 72:87], type="l", lty=1, lwd=2, ylab="RFU", xlab="Cycle")
mtext("E", cex = 1.8, side = 3, adj = 0, font = 2)

# Human NRAS gene, detected with a hydrolysis probe.
matplot(data[, 1], data[, 88:95], type="l", lty=1, lwd=2, ylab="RFU", xlab="Cycle")
mtext("F", cex = 1.8, side = 3, adj = 0, font = 2)

# Water control, detected with a hydrolysis probe.
matplot(data[, 1], data[, 96:97], type="l", lty=1, lwd=2, ylab="RFU", xlab="Cycle")
mtext("G", cex = 1.8, side = 3, adj = 0, font = 2)
```

Printout of all measured samples, their rating by two humans (rater 1 and rater 2) with their dichotomous ratings (0, no hook; 1, hook) and their sources.

- The boggy data (qpcR::boggy) set was taken from the `qpcR` package (Ritz and Spiess, 2008; Spiess *et al.*, 2008).
- The C127EGHP data (chipPCR::C127EGHP) set was taken from the `chipPCR` package (Rödiger *et al.*, 2015b).
- The testdat data (qpcR::testdat) set was taken from the `qpcR` package (Ritz and Spiess, 2008; Spiess *et al.*, 2008).
- Other data were prepared by Evrogen laboratory experiments.

4. Results for the analysis with hookreg() and hookregNL()

This section contains the results of the analysis of the amplification curve data with the **hookreg()** function and the **hookregNL()** function. As in the previous sections, all code was commented to make it reproducible. Some rows in Table 2 and Table 3 appear to be empty. This expected behaviour may occur in cases where the corresponding functions were not able to calculate the coefficients due to a failed model fit or violation of the truncation criterion.

4.1. Results for the analysis of the `hookreg.rdml` data set with hookreg() .

The following code was used to analyze the `hookreg.rdml` data set with **hookreg()** function. The **hookreg()** function fits a linear model to a region of interest. The linear model is used to decide if the amplification curve as a hook effect or hook effect-like curvature.

Stefan Rödiger (stefan.roediger@b-tu.de).

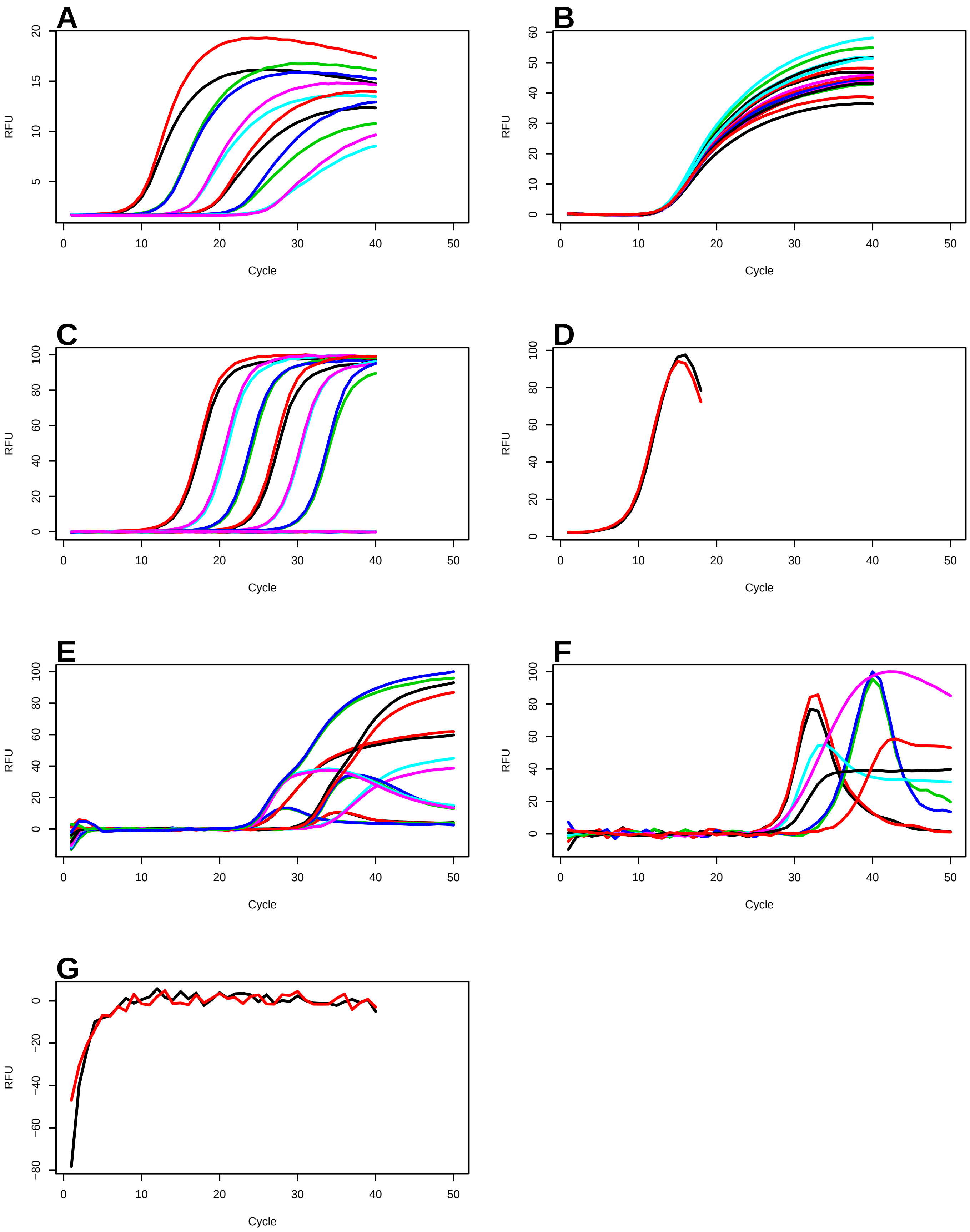

Figure 1. Amplification curves. A) Synthetic template, detected with Syto-13. B) Human *MLC-2v*, detected with a hydrolysis probe. C) *S27a* housekeeping gene, detected with SybrGreen I. D) Whole genome amplification, detected with EvaGreen. E) Human *BRCA1* gene, detected with a hydrolysis probe. F) Human *NRAS* gene, detected with a hydrolysis probe. G) Water control, detected with a hydrolysis probe. See Table 1 for details. RFU, relative fluorescence units.

```r
# Load PCRedux package to obtain the data and make the hookreg() function
# available.
library(PCRedux)
# Load the magrittr to use the %>% pipe-operator
library(magrittr)

# ‘data‘ is a temporary data frame of the hook.rdml amplification curve data file.
# Apply the hookreg() function over the amplification curves and arrange the
# results in the data frame ‘res_hookreg‘.

res_hookreg <- sapply(2L:ncol(data), function(i) {
    hookreg(x=data[, 1], y=data[, i])
}) %>% t %>% data.frame(sample=colnames(data)[-1],.)

# Fetch the calculated parameters from the calculations with the hookreg()
# function as a table ‘res_hookreg_table‘.

res_hookreg_table <- data.frame(sample=as.character(res_hookreg[["sample"]]),
                                intercept=signif(res_hookreg[["intercept"]], 2),
                                slope=signif(res_hookreg[["slope"]], 1),
                                hook.start=signif(res_hookreg[["hook.start"]], 0),
                                hook.delta=signif(res_hookreg[["hook.delta"]], 0),
                                p.value=signif(res_hookreg[["p.value"]], 4),
                                CI.low=signif(res_hookreg[["CI.low"]], 2),
                                CI.up=signif(res_hookreg[["CI.up"]], 2),
                                hook.fit=res_hookreg[["hook.fit"]],
                                hook.CI=res_hookreg[["hook.CI"]],
                                hook=res_hookreg[["hook"]]
)
```

Finally a pretty printout (Table 2) of the results from the **hookreg()** function for the `hookreg.rdml` data set with the following code was prepared.

```r
# Load the xtable to create a LaTeX table from the ‘res_hookreg_table‘.
library(xtable)
options(xtable.comment=FALSE)
print(xtable(res_hookreg_table,
             caption = "Results from the hookreg() function for the hookreg.rdml
             data set.",
             label='res_hookreg_table'),
      size = "normalsize",
      include.rownames = FALSE,
      include.colnames = TRUE,
      caption.placement = "top",
      comment=FALSE,
      table.placement = "!ht", scalebox='0.65'
)
```

The results of the **hookreg()** function are fairly comprehensive. The meaning of the columns is as followed:

- *intercept*, is the intercept from the start of the potential hook to the end of the amplification curve.
- *slope* is the slope from the start of the potential hook to the end of the amplification curve. A negative slope is indicative for a hook effect.
- *hook.start* is the estimated starting cycle of the hook region.
- *hook.delta* is the number of cycles from the *hook.start* to the end of the amplification curve.
- *p.value* describes the significant relationship between the variables in the linear regression model.
- *CI.low* and *CI.up* is the confidence interval (low and up) for the slope parameters in the fitted linear model.
- *hook.fit* is a logical parameter indicating if the fit is significant at a default threshold of 0.005.

- *hook.CI* is a logical parameter indicating if the slope of fitted linear model is within the confidence interval (0.995).

- *hook* is a logical parameter, which combines the significance test and confidence interval test (negative slope).

4.2. Results for the analysis of the `hookreg.rdml` data set with hookregNL() . The following code was used to analyze the `hookreg.rdml` data set with **hookregNL()** function. The procedure is similar to the analysis with the **hookreg()** function.

The **hookreg()** function fits a six parameter sigmoidal model to amplification curve. The non-linear model

$$f(x) = c + k \cdot x + \frac{d - c}{(1 + exp(b(log(x) - log(e))))^f}$$

is used to decide, based on the *k* parameter, if the amplification curve as a hook effect or hook effect-like curvature.

```
# Note that the PCRedux package and the magrittr package need to be loaded (see above).
# Load the qpcR package to prevent messages during the start.
suppressMessages(library(qpcR))

# ‘data‘ is a temporary data frame of the hook.rdml amplification curve data file.
# Apply the hookregNL() function over the amplification curves and arrange the
# results in the data frame ‘res_hookregNL‘.
# Not that ‘suppressMessages()‘ to prevent warning messages from the qpcR package.

res_hookregNL <- suppressMessages(sapply(2L:ncol(data), function(i) {
    hookregNL(x=data[, 1], y=data[, i])
}) %>% t %>% data.frame(sample=colnames(data)[-1],.))

res_hookregNL_table <- data.frame(sample=as.character(res_hookregNL[["sample"]]),
                                  slope=signif(as.numeric(res_hookregNL[["slope"]]), 1),
                                  CI.low=signif(as.numeric(res_hookregNL[["CI.low"]]), 2),
                                  CI.up=signif(as.numeric(res_hookregNL[["CI.up"]]), 2),
                                  hook.CI=unlist(res_hookregNL[["hook"]])
)
```

Finally we prepare a pretty printout (Table 3) of the results from the **hookregNL()** function for the `hookreg.rdml` data set with the following code with the code shown next.

The results of the **hookregNL()** function are less comprehensive then from the **hookreg()** function . The meaning of the columns is as followed:

- *slope* is the slope from the start of the potential hook to the end of the amplification curve that was fitted by a six parameter model. A negative slope is indicative for a hook effect.

- *CI.low* and *CI.up* is the confidence interval (low and up) for the slope parameters in the fitted linear model.

- *hook* is a logical parameter, which combines the significance test and confidence interval test (negative slope).

```
library(xtable)
options(xtable.comment=FALSE)

print(xtable(res_hookregNL_table,
             caption = "Results from the hookregNL() function for the
             hookreg.rdml data set.",
             label='res_hookregNL_table'),
      size = "normalsize",
      include.rownames = FALSE,
      include.colnames = TRUE,
      caption.placement = "top",
      comment=FALSE,
      table.placement = "!ht", scalebox='0.65'
)
```

5. Comparison of the hookreg() and hookregNL() methods

The decisions from the human classification (see Table 1) and the results from the machine decision (section 4.1 and section 4.2) were aggregated in Table 4.

Finally a pretty printout (Table 4) of the aggregated data set with the following code was prepared:

```
# A simple logic was applied to improve the classification result. In this case
# the assumption was, that an amplification curve has an hook effect or hook effect-like
# curvature, if either the hookreg() or hookregNL() function are positive.

meta_hookreg <- sapply(1:nrow(res), function(i){
    ifelse(res[i, "hookreg"] == 1 || res[i, "hookregNL"] == 1, 1, 0)
})

res_out <- data.frame(Sample=res[["Sample"]], res[["Human rater"]],
                      res_hookreg[["hook"]], res_hookregNL_table[["hook.CI"]],
                      meta_hookreg)

colnames(res_out) <- c("Sample",
                       "Human rater",
                       "hookreg",
                       "hookregNL",
                       "hookreg and hoohkreNL combined"
)
```

```
library(xtable)
options(xtable.comment=FALSE)

print(xtable(res_out, digits=0,
             caption = "Aggregated decisions from the human classification and
the results from the machine decision of the hookreg() and hookregNL()
functions.", label='method_comparision'), ,
      caption.placement = "top",
      scalebox='0.65')
```

The performance of the **hookreg()** and **hookregNL()** functions was analyzed with the **performeR()** function of the `PCRedux` package (Table 5). The methods were adopted from Brenner and Gefeller (1997) and Kuhn (2008). Note that the formula for the calculations of the sensitivity, specificity, precision, Negative predictive value, fall-out, alse negative rate, false discovery rate, Accuracy, F1 score, Matthews correlation coefficient and kappa by Cohen are described in the documentation of the `PCRedux` package.

```
res_performeR <- rbind(
    hookreg=performeR(res_out[["hookreg"]], res_out[["Human rater"]]),
                       hookregNL=performeR(res_out[["hookregNL"]], res_out[["Human rater"]]),
                       combined_hookreg=performeR(res_out[["hookreg and hoohkreNL combined"]],
                                                  res_out[["Human rater"]])
) %>% t %>% signif(4)

colnames(res_performeR) <- c("hookreg", "hookregNL", "hookreg and hookregNL")
```

```
library(xtable)
options(xtable.comment=FALSE)

print(xtable(res_performeR, digits=4,
             caption = "Analysis of the performance of both algorithms. The
performance of the individual test and the combination of the tests is shown.
Note that the classification improved if the hookreg() and hookregNL() function
were combined by a logical statement. The measure were determined with the
\\textit{performeR()} function from the \\texttt{PCRedux} package. Sensitivity,
TPR; Specificity, SPC; Precision, PPV; Negative predictive value, NPV; Fall-out,
FPR; False negative rate, FNR; False discovery rate, FDR; Accuracy, ACC; F1
score, F1; Matthews correlation coefficient, MCC, Cohen's kappa (binary
classification), $\\kappa$", label='res_performeR'),
```

```
        size = "normalsize",
        include.rownames = TRUE,
        include.colnames = TRUE,
        caption.placement = "top",
        comment=FALSE,
        table.placement = "!ht", scalebox='0.75'
)
```

6. Hook effect analysis with the rdmlEdit() GUI

A more visual analysis is provided with a comprehensive GUI by installing the `RDML` package and running **rdmlEdit()** as a local server, or alternatively starting rdmlEdit app at the Evrogen server (http://shtest.evrogen.net/rdmlEdit/).

After opening **rdmlEdit**, several tabs at the top of the screen appear. Inside the *Files* tab, click the *Browse...* button to select your file, which is then opened to display its structure on the right of the screen as a dendrogram (Figure 2).

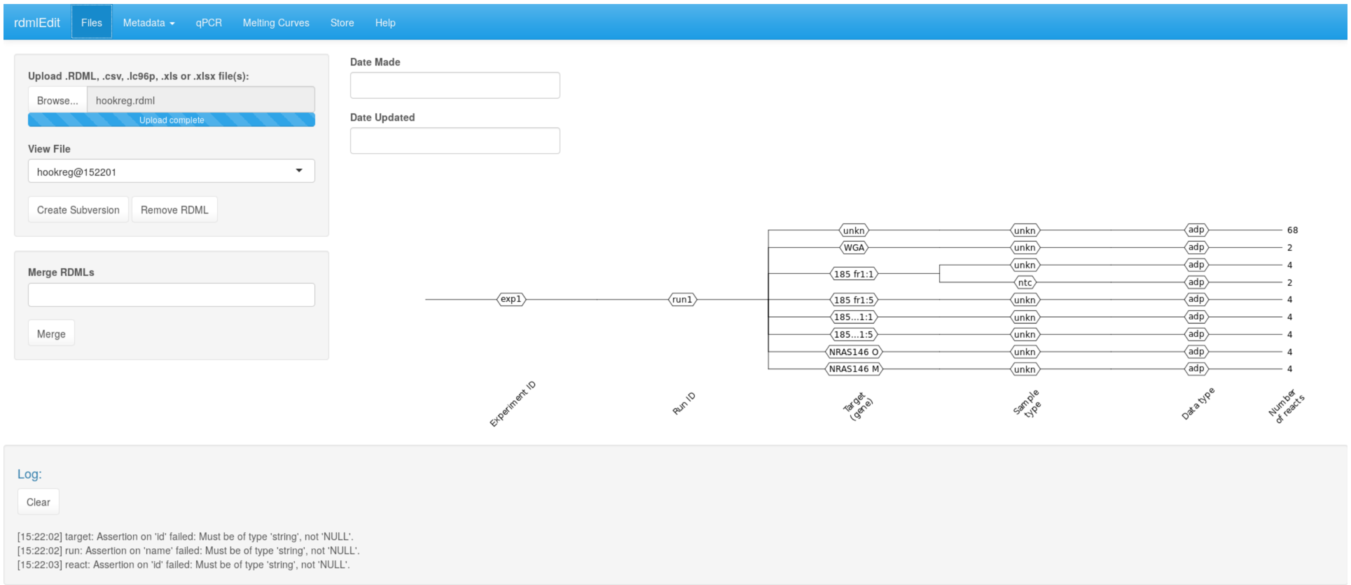

Figure 2. Screenshot of rdmlEdit app dendrogram. The RDML file 'hook.rdml' was loaded in the rdmlEdit app and the dendrogram presented for visualization of the data.

Then, click to the `qPCR` tab to start the analysis (Figure 3). Select a hook detection method from the *Hook Detection Method* selector and wait for the analysis to finish. Results can be viewed inside the table (*hook* column) or by color and line type of PCR curves (select *Hook* at *Color by* and *Line Type by* selectors).

7. Funding

This work was funded by the Federal Ministry of Education and Research (BMBF) InnoProfile-Transfer-Project 03IPT611X and in part by "digilog: Digitale und analoge Begleiter für eine alternde Bevölkerung" (Gesundheitscampus Brandenburg, Brandenburg Ministry for Science, Research and Culture).

Acknowledgments. We thank Franziska Dinter (BTU) for reevaluation of the amplification curve data and Maria Tokarenko (Evrogen JSC) for wet lab experiments conduction.

References

Barratt K, Mackay JF (2002). "Improving Real-Time PCR Genotyping Assays by Asymmetric Amplification." *Journal of Clinical Microbiology*, **40**(4), 1571–1572. ISSN 0095-1137, 1098-660X. . URL http://jcm.asm.org/content/40/4/1571.

Brenner H, Gefeller O (1997). "Variation of sensitivity, specificity, likelihood ratios and predictive values with disease prevalence." *Statistics in medicine*, **16**(9), 981–991. URL http://www.floppybunny.org/robin/web/virtualclassroom/stats/basics/articles/odds_risks/odds_sensitivity)likelihood_ratios_validity_brenner_1997.pdf.

Dvinge H, Bertone P (2009). "HTqPCR: high-throughput analysis and visualization of quantitative real-time PCR data in R." *Bioinformatics*, **25**(24), 3325–3326. ISSN 1367-4803, 1460-2059. . URL http://bioinformatics.oxfordjournals.org/content/25/24/3325.

Kemperman L, McCall MN (2017). "miRcomp-Shiny: Interactive assessment of qPCR-based microRNA quantification and quality control algorithms." *F1000Research*, **6**, 2046. ISSN 2046-1402. URL https://f1000research.com/articles/6-2046/v1.

Kuhn M (2008). "Building Predictive Models in R Using the caret Package." *Journal of Statistical Software*, **28**(5). ISSN 1548-7660. URL http://www.jstatsoft.org/v28/i05/.

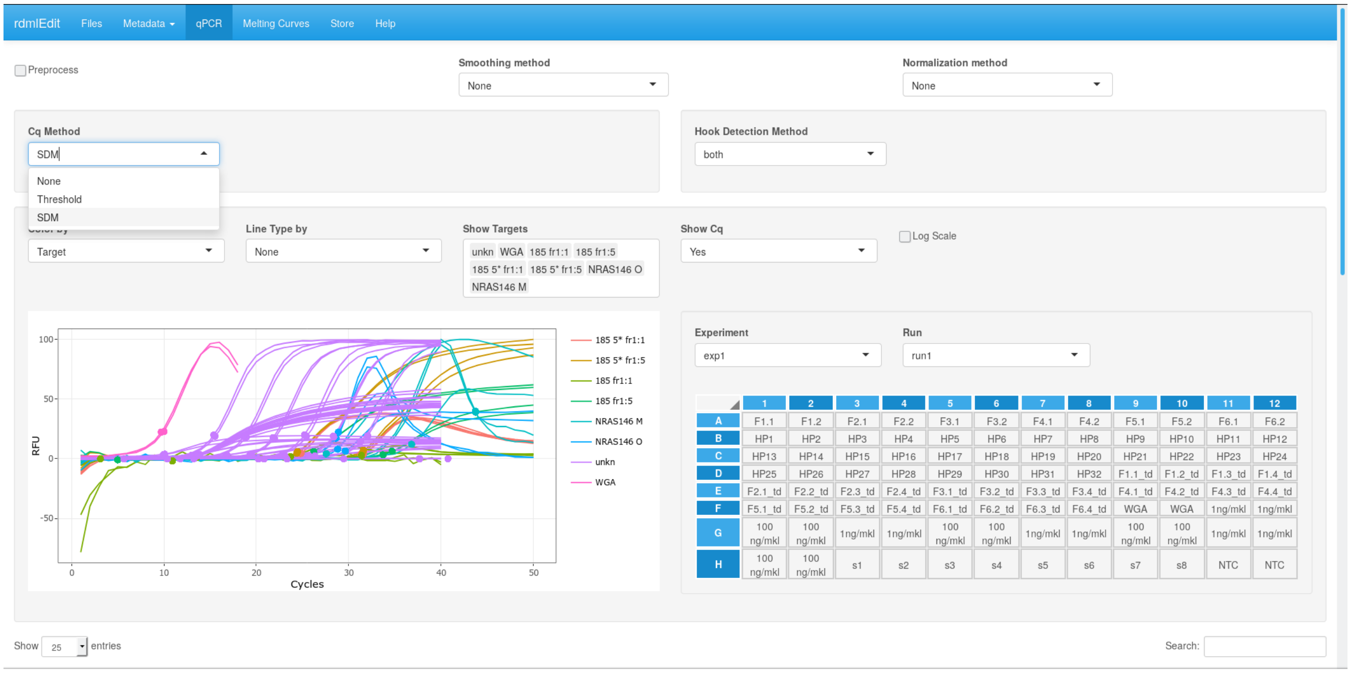

Figure 3. Screenshot of rdmlEdit app. The RDML file 'hook.rdml' was loaded in the rdmlEdit app and the visual appearance in the qPCR tab as adjusted as needed.

Lefever S, Hellemans J, Pattyn F, Przybylski DR, Taylor C, Geurts R, Untergasser A, Vandesompele J, Consortium obotR (2009). "RDML: structured language and reporting guidelines for real-time quantitative PCR data." *Nucleic Acids Research*, **37**(7), 2065–2069. ISSN 0305-1048, 1362-4962. . 00097, URL http://nar.oxfordjournals.org/content/37/7/2065.

Martins C, Lima G, Carvalho M, Cainé L, Porto M (2015). "DNA quantification by real-time PCR in different forensic samples." *Forensic Science International: Genetics Supplement Series*, **5**, e545–e546. ISSN 18751768. . URL http://linkinghub.elsevier.com/retrieve/pii/S1875176815301335.

R Core Team (2017). *R: A Language and Environment for Statistical Computing*. R Foundation for Statistical Computing, Vienna, Austria. URL https://www.R-project.org/.

Ritz C, Spiess AN (2008). "qpcR: an R package for sigmoidal model selection in quantitative real-time polymerase chain reaction analysis." *Bioinformatics*, **24**(13), 1549–1551. ISSN 1367-4803, 1460-2059. . URL http://bioinformatics.oxfordjournals.org/content/24/13/1549.

Rödiger S, Burdukiewicz M, Blagodatskikh KA, Schierack P (2015a). "R as an Environment for the Reproducible Analysis of DNA Amplification Experiments." *The R Journal*, **7**(2), 127–150. URL http://journal.r-project.org/archive/2015-1/RJ-2015-1.pdf.

Rödiger S, Burdukiewicz M, Schierack P (2015b). "chipPCR: an R package to pre-process raw data of amplification curves." *Bioinformatics*, **31**(17), 2900–2902. ISSN 1367-4803, 1460-2059. . URL http://bioinformatics.oxfordjournals.org/content/31/17/2900.

Rödiger S, Burdukiewicz M, Spiess AN, Blagodatskikh K (2017). "Enabling reproducible real-time quantitative PCR research: the RDML package." *Bioinformatics*. . URL https://academic.oup.com/bioinformatics/article/doi/10.1093/bioinformatics/btx528/4095640/Enabling-reproducible-real-time-quantitative-PCR.

Rödiger S, Friedrichsmeier T, Kapat P, Michalke M (2012). "RKWard: a comprehensive graphical user interface and integrated development environment for statistical analysis with R." *Journal of Statistical Software*, **49**(9), 1–34. URL https://www.jstatsoft.org/article/view/v049i09/v49i09.pdf.

Ronde MWJd, Ruijter JM, Lanfear D, Bayes-Genis A, Kok MGM, Creemers EE, Pinto YM, Pinto-Sietsma SJ (2017). "Practical data handling pipeline improves performance of qPCR-based circulating miRNA measurements." *RNA*, **23**(5), 811–821. ISSN 1355-8382, 1469-9001. . URL http://rnajournal.cshlp.org/content/23/5/811.

Ruijter JM, Lefever S, Anckaert J, Hellemans J, Pfaffl MW, Benes V, Bustin SA, Vandesompele J, Untergasser A, Consortium obotR (2015). "RDML-Ninja and RDMLdb for standardized exchange of qPCR data." *BMC Bioinformatics*, **16**(1), 197. ISSN 1471-2105. .

Ruijter JM, Pfaffl MW, Zhao S, Spiess AN, Boggy G, Blom J, Rutledge RG, Sisti D, Lievens A, De Preter K, Derveaux S, Hellemans J, Vandesompele J (2013). "Evaluation of qPCR curve analysis methods for reliable biomarker discovery: Bias, resolution, precision, and implications." *Methods*, **59**(1), 32–46. ISSN 10462023. . URL http://linkinghub.elsevier.com/retrieve/pii/S1046202312002290.

Sauer E, Reinke AK, Courts C (2016). "Differentiation of five body fluids from forensic samples by expression analysis of four microRNAs using quantitative PCR." *Forensic Science International: Genetics*, **22**, 89–99. ISSN 18724973. . URL http://linkinghub.elsevier.com/retrieve/pii/S1872497316300187.

Spiess AN, Deutschmann C, Burdukiewicz M, Himmelreich R, Klat K, Schierack P, Rödiger S (2015). "Impact of Smoothing on Parameter Estimation in Quantitative DNA Amplification Experiments." *Clinical Chemistry*, **61**(2), 379–388. ISSN 0009-9147, 1530-8561. .

Spiess AN, Feig C, Ritz C (2008). "Highly accurate sigmoidal fitting of real-time PCR data by introducing a parameter for asymmetry." *BMC Bioinformatics*, **9**(1), 221. ISSN 1471-2105. . URL http://www.biomedcentral.com/1471-2105/9/221/abstract.

Spiess AN, Rödiger S, Burdukiewicz M, Volksdorf T, Tellinghuisen J (2016). "System-specific periodicity in quantitative real-time polymerase chain reaction data questions threshold-based quantitation." *Scientific Reports*, **6**, 38951. ISSN 2045-2322. . URL http://www.nature.com/articles/srep38951.

Table 1. Overview of the used amplification curve data. The samples names, data source (origin of data either from an existing data set or prepared for this study), the detection chemistries (intercalator (Syto-13, SyberGreenI, EvaGreen), hydrolysis probes (TaqMan (Cy5/BHQ2) , TaqMan (HEX/BHQ1))) and calculations by tow humans.

#	Sample	Data Source	Target	Chemistry	Hook effect-like Rater 1	Hook effect-like Rater 2	Rating Conformity
1	F1.1	qpcR::boggy	synthetic template	Syto-13	1	1	1
2	F1.2	qpcR::boggy	synthetic template	Syto-13	1	1	1
3	F2.1	qpcR::boggy	synthetic template	Syto-13	1	1	1
4	F2.2	qpcR::boggy	synthetic template	Syto-13	1	1	1
5	F3.1	qpcR::boggy	synthetic template	Syto-13	0	0	1
6	F3.2	qpcR::boggy	synthetic template	Syto-13	0	0	1
7	F4.1	qpcR::boggy	synthetic template	Syto-13	0	0	1
8	F4.2	qpcR::boggy	synthetic template	Syto-13	0	0	1
9	F5.1	qpcR::boggy	synthetic template	Syto-13	0	0	1
10	F5.2	qpcR::boggy	synthetic template	Syto-13	0	0	1
11	F6.1	qpcR::boggy	synthetic template	Syto-13	0	0	1
12	F6.2	qpcR::boggy	synthetic template	Syto-13	0	0	1
13	HP1	chipPCR::C127EGHP	MLC-2v	TaqMan (Cy5/BHQ2)	0	0	1
14	HP2	chipPCR::C127EGHP	MLC-2v	TaqMan (Cy5/BHQ2)	0	0	1
15	HP3	chipPCR::C127EGHP	MLC-2v	TaqMan (Cy5/BHQ2)	0	0	1
16	HP4	chipPCR::C127EGHP	MLC-2v	TaqMan (Cy5/BHQ2)	0	0	1
17	HP5	chipPCR::C127EGHP	MLC-2v	TaqMan (Cy5/BHQ2)	0	0	1
18	HP6	chipPCR::C127EGHP	MLC-2v	TaqMan (Cy5/BHQ2)	0	0	1
19	HP7	chipPCR::C127EGHP	MLC-2v	TaqMan (Cy5/BHQ2)	0	0	1
20	HP8	chipPCR::C127EGHP	MLC-2v	TaqMan (Cy5/BHQ2)	0	0	1
21	HP9	chipPCR::C127EGHP	MLC-2v	TaqMan (Cy5/BHQ2)	0	0	1
22	HP10	chipPCR::C127EGHP	MLC-2v	TaqMan (Cy5/BHQ2)	0	0	1
23	HP11	chipPCR::C127EGHP	MLC-2v	TaqMan (Cy5/BHQ2)	0	0	1
24	HP12	chipPCR::C127EGHP	MLC-2v	TaqMan (Cy5/BHQ2)	0	0	1
25	HP13	chipPCR::C127EGHP	MLC-2v	TaqMan (Cy5/BHQ2)	0	0	1
26	HP14	chipPCR::C127EGHP	MLC-2v	TaqMan (Cy5/BHQ2)	0	0	1
27	HP15	chipPCR::C127EGHP	MLC-2v	TaqMan (Cy5/BHQ2)	0	0	1
28	HP16	chipPCR::C127EGHP	MLC-2v	TaqMan (Cy5/BHQ2)	0	0	1
29	HP17	chipPCR::C127EGHP	MLC-2v	TaqMan (Cy5/BHQ2)	0	0	1
30	HP18	chipPCR::C127EGHP	MLC-2v	TaqMan (Cy5/BHQ2)	0	0	1
31	HP19	chipPCR::C127EGHP	MLC-2v	TaqMan (Cy5/BHQ2)	0	0	1
32	HP20	chipPCR::C127EGHP	MLC-2v	TaqMan (Cy5/BHQ2)	0	0	1
33	HP21	chipPCR::C127EGHP	MLC-2v	TaqMan (Cy5/BHQ2)	0	0	1
34	HP22	chipPCR::C127EGHP	MLC-2v	TaqMan (Cy5/BHQ2)	0	0	1
35	HP23	chipPCR::C127EGHP	MLC-2v	TaqMan (Cy5/BHQ2)	0	0	1
36	HP24	chipPCR::C127EGHP	MLC-2v	TaqMan (Cy5/BHQ2)	0	0	1
37	HP25	chipPCR::C127EGHP	MLC-2v	TaqMan (Cy5/BHQ2)	0	0	1
38	HP26	chipPCR::C127EGHP	MLC-2v	TaqMan (Cy5/BHQ2)	0	0	1
39	HP27	chipPCR::C127EGHP	MLC-2v	TaqMan (Cy5/BHQ2)	0	0	1
40	HP28	chipPCR::C127EGHP	MLC-2v	TaqMan (Cy5/BHQ2)	0	0	1
41	HP29	chipPCR::C127EGHP	MLC-2v	TaqMan (Cy5/BHQ2)	0	0	1
42	HP30	chipPCR::C127EGHP	MLC-2v	TaqMan (Cy5/BHQ2)	0	0	1
43	HP31	chipPCR::C127EGHP	MLC-2v	TaqMan (Cy5/BHQ2)	0	0	1
44	HP32	chipPCR::C127EGHP	MLC-2v	TaqMan (Cy5/BHQ2)	0	0	1
45	F1.1_td	qpcR::testdat	S27a housekeeping gene	SybrGreen I	0	0	1
46	F1.2_td	qpcR::testdat	S27a housekeeping gene	SybrGreen I	0	0	1
47	F1.3_td	qpcR::testdat	S27a housekeeping gene	SybrGreen I	0	0	1
48	F1.4_td	qpcR::testdat	S27a housekeeping gene	SybrGreen I	0	0	1
49	F2.1_td	qpcR::testdat	S27a housekeeping gene	SybrGreen I	0	0	1
50	F2.2_td	qpcR::testdat	S27a housekeeping gene	SybrGreen I	0	0	1
51	F2.3_td	qpcR::testdat	S27a housekeeping gene	SybrGreen I	0	0	1
52	F2.4_td	qpcR::testdat	S27a housekeeping gene	SybrGreen I	0	0	1
53	F3.1_td	qpcR::testdat	S27a housekeeping gene	SybrGreen I	0	0	1
54	F3.2_td	qpcR::testdat	S27a housekeeping gene	SybrGreen I	0	0	1
55	F3.3_td	qpcR::testdat	S27a housekeeping gene	SybrGreen I	0	0	1
56	F3.4_td	qpcR::testdat	S27a housekeeping gene	SybrGreen I	0	0	1
57	F4.1_td	qpcR::testdat	S27a housekeeping gene	SybrGreen I	0	0	1
58	F4.2_td	qpcR::testdat	S27a housekeeping gene	SybrGreen I	0	0	1
59	F4.3_td	qpcR::testdat	S27a housekeeping gene	SybrGreen I	0	0	1
60	F4.4_td	qpcR::testdat	S27a housekeeping gene	SybrGreen I	0	0	1
61	F5.1_td	qpcR::testdat	S27a housekeeping gene	SybrGreen I	0	0	1
62	F5.2_td	qpcR::testdat	S27a housekeeping gene	SybrGreen I	0	0	1
63	F5.3_td	qpcR::testdat	S27a housekeeping gene	SybrGreen I	0	0	1
64	F5.4_td	qpcR::testdat	S27a housekeeping gene	SybrGreen I	0	0	1
65	F6.1_td	qpcR::testdat	S27a housekeeping gene	SybrGreen I	0	0	1
66	F6.2_td	qpcR::testdat	S27a housekeeping gene	SybrGreen I	0	0	1
67	F6.3_td	qpcR::testdat	S27a housekeeping gene	SybrGreen I	0	0	1
68	F6.4_td	qpcR::testdat	S27a housekeeping gene	SybrGreen I	0	0	1
69	F09˜WGA	Evrogen lab experiment	Whole genome amplification	EvaGreen	1	1	1
70	F10˜WGA	Evrogen lab experiment	Whole genome amplification	EvaGreen	1	1	1
71	F11˜1ng/mkl	Evrogen lab experiment	BRCA1 gene	TaqMan (HEX/BHQ1)	1	1	1
72	F12˜1ng/mkl	Evrogen lab experiment	BRCA1 gene	TaqMan (HEX/BHQ1)	1	1	1
73	G01˜100ng/mkl	Evrogen lab experiment	BRCA1 gene	TaqMan (HEX/BHQ1)	1	1	1
74	G02˜100ng/mkl	Evrogen lab experiment	BRCA1 gene	TaqMan (HEX/BHQ1)	1	1	1
75	G03˜1ng/mkl	Evrogen lab experiment	BRCA1 gene	TaqMan (HEX/BHQ1)	0	0	1
76	G04˜1ng/mkl	Evrogen lab experiment	BRCA1 gene	TaqMan (HEX/BHQ1)	0	0	1
77	G05˜100ng/mkl	Evrogen lab experiment	BRCA1 gene	TaqMan (HEX/BHQ1)	0	0	1
78	G06˜100ng/mkl	Evrogen lab experiment	BRCA1 gene	TaqMan (HEX/BHQ1)	0	0	1
79	G07˜1ng/mkl	Evrogen lab experiment	BRCA1 gene	TaqMan (HEX/BHQ1)	1	1	1
80	G08˜1ng/mkl	Evrogen lab experiment	BRCA1 gene	TaqMan (HEX/BHQ1)	1	1	1
81	G09˜100ng/mkl	Evrogen lab experiment	BRCA1 gene	TaqMan (HEX/BHQ1)	1	1	1
82	G10˜100ng/mkl	Evrogen lab experiment	BRCA1 gene	TaqMan (HEX/BHQ1)	1	1	1
83	G11˜1ng/mkl	Evrogen lab experiment	BRCA1 gene	TaqMan (HEX/BHQ1)	0	0	1
84	G12˜1ng/mkl	Evrogen lab experiment	BRCA1 gene	TaqMan (HEX/BHQ1)	0	0	1
85	H01˜100ng/mkl	Evrogen lab experiment	BRCA1 gene	TaqMan (HEX/BHQ1)	0	0	1
86	H02˜100ng/mkl	Evrogen lab experiment	BRCA1 gene	TaqMan (HEX/BHQ1)	0	0	1
87	s1	Evrogen lab experiment	NRAS gene	TaqMan (FAM/BHQ1)	1	1	1
88	s2	Evrogen lab experiment	NRAS gene	TaqMan (FAM/BHQ1)	1	1	1
89	s3	Evrogen lab experiment	NRAS gene	TaqMan (FAM/BHQ1)	1	1	1
90	s4	Evrogen lab experiment	NRAS gene	TaqMan (FAM/BHQ1)	1	1	1
91	s5	Evrogen lab experiment	NRAS gene	TaqMan (FAM/BHQ1)	1	1	1
92	s6	Evrogen lab experiment	NRAS gene	TaqMan (FAM/BHQ1)	1	1	1
93	s7	Evrogen lab experiment	NRAS gene	TaqMan (FAM/BHQ1)	0	0	1
94	s8	Evrogen lab experiment	NRAS gene	TaqMan (FAM/BHQ1)	1	1	1
95	NTC	Evrogen lab experiment	NRAS gene	TaqMan (FAM/BHQ1)	0	0	1
96	NTC	Evrogen lab experiment	NRAS gene	TaqMan (FAM/BHQ1)	0	0	1

Table 2. Results from the hookreg() function for the hookreg.rdml data set.

sample	intercept	slope	hook.start	hook.delta	p.value	CI.low	CI.up	hook.fit	hook.CI	hook
A01˜F1.1	1.20	-0.01	30.00	20.00	0.00	1.20	-0.01	1.00	1.00	1.00
A02˜F1.2	1.20	-0.01	30.00	20.00	0.00	1.20	-0.01	1.00	1.00	1.00
A03˜F2.1	1.20	-0.01	30.00	9.00	0.00	1.20	-0.00	1.00	1.00	1.00
A04˜F2.2	1.20	-0.01	30.00	9.00	0.00	1.20	-0.00	1.00	1.00	1.00
A05˜F3.1	1.10	-0.00	40.00	6.00	0.05	1.20	0.00	0.00	0.00	0.00
A06˜F3.2	1.10	-0.00	40.00	6.00	0.02	1.20	0.00	0.00	0.00	0.00
A07˜F4.1								0.00	0.00	0.00
A08˜F4.2								0.00	0.00	0.00
A09˜F5.1								0.00	0.00	0.00
A10˜F5.2								0.00	0.00	0.00
A11˜F6.1								0.00	0.00	0.00
A12˜F6.2								0.00	0.00	0.00
B01˜HP1								0.00	0.00	0.00
B02˜HP2								0.00	0.00	0.00
B03˜HP3								0.00	0.00	0.00
B04˜HP4								0.00	0.00	0.00
B05˜HP5								0.00	0.00	0.00
B06˜HP6								0.00	0.00	0.00
B07˜HP7								0.00	0.00	0.00
B08˜HP8								0.00	0.00	0.00
B09˜HP9								0.00	0.00	0.00
B10˜HP10								0.00	0.00	0.00
B11˜HP11								0.00	0.00	0.00
B12˜HP12								0.00	0.00	0.00
C01˜HP13								0.00	0.00	0.00
C02˜HP14								0.00	0.00	0.00
C03˜HP15								0.00	0.00	0.00
C04˜HP16								0.00	0.00	0.00
C05˜HP17								0.00	0.00	0.00
C06˜HP18								0.00	0.00	0.00
C07˜HP19								0.00	0.00	0.00
C08˜HP20								0.00	0.00	0.00
C09˜HP21								0.00	0.00	0.00
C10˜HP22								0.00	0.00	0.00
C11˜HP23								0.00	0.00	0.00
C12˜HP24								0.00	0.00	0.00
D01˜HP25								0.00	0.00	0.00
D02˜HP26								0.00	0.00	0.00
D03˜HP27								0.00	0.00	0.00
D04˜HP28								0.00	0.00	0.00
D05˜HP29								0.00	0.00	0.00
D06˜HP30								0.00	0.00	0.00
D07˜HP31								0.00	0.00	0.00
D08˜HP32								0.00	0.00	0.00
D09˜F1.1_td	1.00	-0.00	30.00	7.00	0.01	1.10	0.00	0.00	0.00	0.00
D10˜F1.2_td	1.10	-0.00	30.00	10.00	0.00	1.10	-0.00	1.00	1.00	1.00
D11˜F1.3_td	0.73	-0.02	10.00	30.00	0.05	1.50	0.01	0.00	0.00	0.00
D12˜F1.4_td	0.08	-0.00	3.00	40.00	0.42	0.40	0.01	0.00	0.00	0.00
E01˜F2.1_td	1.00	-0.00	30.00	7.00	0.04	1.10	0.00	0.00	0.00	0.00
E02˜F2.2_td	1.10	-0.00	40.00	5.00	0.15	1.30	0.00	0.00	0.00	0.00
E03˜F2.3_td	-0.13	0.00	20.00	20.00	0.90	1.40	0.06	0.00	0.00	0.00
E04˜F2.4_td	3.10	-0.08	30.00	8.00	0.24	12.00	0.17	0.00	0.00	0.00
E05˜F3.1_td								0.00	0.00	0.00
E06˜F3.2_td								0.00	0.00	0.00
E07˜F3.3_td	0.55	-0.02	10.00	30.00	0.09	1.40	0.01	0.00	0.00	0.00
E08˜F3.4_td	0.11	-0.00	10.00	30.00	0.84	1.00	0.03	0.00	0.00	0.00
E09˜F4.1_td								0.00	0.00	0.00
E10˜F4.2_td								0.00	0.00	0.00
E11˜F4.3_td	2.90	-0.08	30.00	10.00	0.14	9.50	0.11	0.00	0.00	0.00
E12˜F4.4_td	0.26	-0.02	6.00	40.00	0.08	1.20	0.01	0.00	0.00	0.00
F01˜F5.1_td								0.00	0.00	0.00
F02˜F5.2_td								0.00	0.00	0.00
F03˜F5.3_td	2.20	-0.06	30.00	10.00	0.06	5.60	0.04	0.00	0.00	0.00
F04˜F5.4_td	-0.08	0.00	20.00	20.00	0.89	1.50	0.06	0.00	0.00	0.00
F05˜F6.1_td								0.00	0.00	0.00
F06˜F6.2_td								0.00	0.00	0.00
F07˜F6.3_td	0.67	-0.02	20.00	20.00	0.24	2.20	0.03	0.00	0.00	0.00
F08˜F6.4_td	0.09	-0.00	4.00	40.00	0.73	0.62	0.02	0.00	0.00	0.00
F09˜WGA								0.00	0.00	0.00
F10˜WGA								0.00	0.00	0.00
F11˜1ng/mkl	2.40	-0.04	40.00	20.00	0.00	3.20	-0.02	1.00	1.00	1.00
F12˜1ng/mkl	2.30	-0.04	40.00	20.00	0.00	3.30	-0.02	1.00	1.00	1.00
G01˜100 ng/mkl	1.60	-0.03	30.00	20.00	0.00	2.10	-0.02	1.00	1.00	1.00
G02˜100 ng/mkl	1.70	-0.03	30.00	20.00	0.00	2.20	-0.02	1.00	1.00	1.00
G03˜1ng/mkl								0.00	0.00	0.00
G04˜1ng/mkl								0.00	0.00	0.00
G05˜100 ng/mkl								0.00	0.00	0.00
G06˜100 ng/mkl								0.00	0.00	0.00
G07˜1ng/mkl	3.00	-0.05	40.00	10.00	0.00	3.30	-0.05	1.00	1.00	1.00
G08˜1ng/mkl	3.00	-0.05	40.00	10.00	0.00	3.30	-0.04	1.00	1.00	1.00
G09˜100 ng/mkl	2.50	-0.04	30.00	20.00	0.00	2.70	-0.04	1.00	1.00	1.00
G10˜100 ng/mkl	2.60	-0.05	30.00	20.00	0.00	2.80	-0.04	1.00	1.00	1.00
G11˜1ng/mkl								0.00	0.00	0.00
G12˜1ng/mkl								0.00	0.00	0.00
H01˜100 ng/mkl								0.00	0.00	0.00
H02˜100 ng/mkl								0.00	0.00	0.00
H03˜s1	2.40	-0.05	30.00	20.00	0.00	3.30	-0.03	1.00	1.00	1.00
H04˜s2	2.20	-0.05	30.00	20.00	0.00	3.20	-0.02	1.00	1.00	1.00
H05˜s3	4.10	-0.08	40.00	10.00	0.00	6.20	-0.04	1.00	1.00	1.00
H06˜s4	4.60	-0.09	40.00	10.00	0.00	6.80	-0.04	1.00	1.00	1.00
H07˜s5	1.60	-0.02	30.00	20.00	0.00	2.10	-0.01	1.00	1.00	1.00
H08˜s6	1.80	-0.02	40.00	9.00	0.00	2.10	-0.01	1.00	1.00	1.00
H09˜s7								0.00	0.00	0.00
H10˜s8	1.50	-0.01	40.00	8.00	0.00	1.90	-0.00	1.00	1.00	1.00
H11˜NTC	0.92	-0.03	10.00	30.00	0.00	1.50	-0.01	1.00	1.00	1.00
H12˜NTC	0.50	-0.01	10.00	30.00	0.19	1.50	0.02	0.00	0.00	0.00

Table 3. Results from the hookregNL() function for the hookreg.rdml data set.

sample	slope	CI.low	CI.up	hook.CI
A01~F1.1	-0.10	-0.16	-0.12	1.00
A02~F1.2	-0.20	-0.19	-0.15	1.00
A03~F2.1	-0.09	-0.13	-0.06	1.00
A04~F2.2	-0.09	-0.12	-0.06	1.00
A05~F3.1	-0.02	-0.05	0.00	0.00
A06~F3.2	-0.02	-0.05	0.01	0.00
A07~F4.1	0.00			0.00
A08~F4.2	0.00	-0.01	0.02	0.00
A09~F5.1	0.01			0.00
A10~F5.2	0.01			0.00
A11~F6.1	0.00			0.00
A12~F6.2	0.00			0.00
B01~HP1	0.01			0.00
B02~HP2	0.08			0.00
B03~HP3	0.06			0.00
B04~HP4	0.03			0.00
B05~HP5	0.04			0.00
B06~HP6	0.02			0.00
B07~HP7	-0.10			0.00
B08~HP8	0.03			0.00
B09~HP9	0.05			0.00
B10~HP10	0.05			0.00
B11~HP11	0.06			0.00
B12~HP12	0.07			0.00
C01~HP13	0.05			0.00
C02~HP14	-0.04			0.00
C03~HP15	0.08			0.00
C04~HP16	0.09			0.00
C05~HP17	0.05			0.00
C06~HP18	0.03			0.00
C07~HP19	0.10			0.00
C08~HP20	0.02			0.00
C09~HP21	0.06			0.00
C10~HP22	0.01			0.00
C11~HP23	0.10			0.00
C12~HP24	0.06			0.00
D01~HP25	0.09			0.00
D02~HP26	0.10			0.00
D03~HP27	0.10			0.00
D04~HP28	0.10			0.00
D05~HP29	0.20			0.00
D06~HP30	0.10			0.00
D07~HP31	0.10			0.00
D08~HP32	0.04			0.00
D09~F1.1_td	0.09	0.02	0.16	0.00
D10~F1.2_td	-0.05			0.00
D11~F1.3_td				0.00
D12~F1.4_td				0.00
E01~F2.1_td	0.10	0.07	0.22	0.00
E02~F2.2_td	0.05			0.00
E03~F2.3_td	-0.00			0.00
E04~F2.4_td				0.00
E05~F3.1_td	0.10	0.07	0.21	0.00
E06~F3.2_td	0.09	0.05	0.14	0.00
E07~F3.3_td	-0.00			0.00
E08~F3.4_td	-0.00			0.00
E09~F4.1_td	0.10	0.03	0.16	0.00
E10~F4.2_td	0.08	0.03	0.13	0.00
E11~F4.3_td	-0.00			0.00
E12~F4.4_td	0.00			0.00
F01~F5.1_td	0.05	0.02	0.09	0.00
F02~F5.2_td	0.05			0.00
F03~F5.3_td	-0.01			0.00
F04~F5.4_td				0.00
F05~F6.1_td	0.03			0.00
F06~F6.2_td	0.03			0.00
F07~F6.3_td				0.00
F08~F6.4_td	-0.04			0.00
F09~WGA	-20.00	-38.00	-9.50	1.00
F10~WGA	-20.00	-32.00	-10.00	1.00
F11~1ng/mkl	-0.40			0.00
F12~1ng/mkl	-0.40			0.00
G01~100 ng/mkl	-0.40			0.00
G02~100 ng/mkl	-0.40			0.00
G03~1ng/mkl	0.02	-0.00	0.03	0.00
G04~1ng/mkl	-0.01			0.00
G05~100 ng/mkl	0.03			0.00
G06~100 ng/mkl	0.10			0.00
G07~1ng/mkl	-1.00			0.00
G08~1ng/mkl	-1.00			0.00
G09~100 ng/mkl	-1.00			0.00
G10~100 ng/mkl	-1.00			0.00
G11~1ng/mkl	-0.03			0.00
G12~1ng/mkl	-0.02			0.00
H01~100 ng/mkl	-0.10			0.00
H02~100 ng/mkl	0.01			0.00
H03~s1	-4.00			0.00
H04~s2	-4.00			0.00
H05~s3	-5.00			0.00
H06~s4	-8.00			0.00
H07~s5	-0.80			0.00
H08~s6	-0.50	-0.88	-0.13	1.00
H09~s7	0.05	0.01	0.08	0.00
H10~s8	-0.04			0.00
H11~NTC	40.00			0.00
H12~NTC	40.00			0.00

Table 4. Aggregated decisions from the human classification and the results from the machine decision of the hookreg() and hookregNL() functions.

	Sample	Human rater	hookreg	hookregNL	hookreg and hoohkreNL combined
1	F1.1	1	1	1	1
2	F1.2	1	1	1	1
3	F2.1	1	1	1	1
4	F2.2	1	1	1	1
5	F3.1	0	0	0	0
6	F3.2	0	0	0	0
7	F4.1	0	0	0	0
8	F4.2	0	0	0	0
9	F5.1	0	0	0	0
10	F5.2	0	0	0	0
11	F6.1	0	0	0	0
12	F6.2	0	0	0	0
13	HP1	0	0	0	0
14	HP2	0	0	0	0
15	HP3	0	0	0	0
16	HP4	0	0	0	0
17	HP5	0	0	0	0
18	HP6	0	0	0	0
19	HP7	0	0	0	0
20	HP8	0	0	0	0
21	HP9	0	0	0	0
22	HP10	0	0	0	0
23	HP11	0	0	0	0
24	HP12	0	0	0	0
25	HP13	0	0	0	0
26	HP14	0	0	0	0
27	HP15	0	0	0	0
28	HP16	0	0	0	0
29	HP17	0	0	0	0
30	HP18	0	0	0	0
31	HP19	0	0	0	0
32	HP20	0	0	0	0
33	HP21	0	0	0	0
34	HP22	0	0	0	0
35	HP23	0	0	0	0
36	HP24	0	0	0	0
37	HP25	0	0	0	0
38	HP26	0	0	0	0
39	HP27	0	0	0	0
40	HP28	0	0	0	0
41	HP29	0	0	0	0
42	HP30	0	0	0	0
43	HP31	0	0	0	0
44	HP32	0	0	0	0
45	F1.1_td	0	0	0	0
46	F1.2_td	0	1	0	1
47	F1.3_td	0	0	0	0
48	F1.4_td	0	0	0	0
49	F2.1_td	0	0	0	0
50	F2.2_td	0	0	0	0
51	F2.3_td	0	0	0	0
52	F2.4_td	0	0	0	0
53	F3.1_td	0	0	0	0
54	F3.2_td	0	0	0	0
55	F3.3_td	0	0	0	0
56	F3.4_td	0	0	0	0
57	F4.1_td	0	0	0	0
58	F4.2_td	0	0	0	0
59	F4.3_td	0	0	0	0
60	F4.4_td	0	0	0	0
61	F5.1_td	0	0	0	0
62	F5.2_td	0	0	0	0
63	F5.3_td	0	0	0	0
64	F5.4_td	0	0	0	0
65	F6.1_td	0	0	0	0
66	F6.2_td	0	0	0	0
67	F6.3_td	0	0	0	0
68	F6.4_td	0	0	0	0
69	F09˜WGA	1	0	1	1
70	F10˜WGA	1	0	1	1
71	F11˜1ng/mkl	1	1	0	1
72	F12˜1ng/mkl	1	1	0	1
73	G01˜100ng/mkl	1	1	0	1
74	G02˜100ng/mkl	1	1	0	1
75	G03˜1ng/mkl	0	0	0	0
76	G04˜1ng/mkl	0	0	0	0
77	G05˜100ng/mkl	0	0	0	0
78	G06˜100ng/mkl	0	0	0	0
79	G07˜1ng/mkl	1	1	0	1
80	G08˜1ng/mkl	1	1	0	1
81	G09˜100ng/mkl	1	1	0	1
82	G10˜100ng/mkl	1	1	0	1
83	G11˜1ng/mkl	0	0	0	0
84	G12˜1ng/mkl	0	0	0	0
85	H01˜100ng/mkl	0	0	0	0
86	H02˜100ng/mkl	0	0	0	0
87	s1	1	1	0	1
88	s2	1	1	0	1
89	s3	1	1	0	1
90	s4	1	1	0	1
91	s5	1	1	0	1
92	s6	1	1	1	1
93	s7	0	0	0	0
94	s8	1	1	0	1
95	NTC	0	1	0	1
96	NTC	0	0	0	0

Table 5. Analysis of the performance of both algorithms. The performance of the individual test and the combination of the tests is shown. Note that the classification improved if the hookreg() and hookregNL() function were combined by a logical statement. The measure were determined with the *performeR()* function from the `PCRedux` package. Sensitivity, TPR; Specificity, SPC; Precision, PPV; Negative predictive value, NPV; Fall-out, FPR; False negative rate, FNR; False discovery rate, FDR; Accuracy, ACC; F1 score, F1; Matthews correlation coefficient, MCC, Cohen's kappa (binary classification), κ

	hookreg	hookregNL	hookreg and hookregNL
TPR	0.9048	0.3333	1.0000
SPC	0.9733	1.0000	0.9733
PPV	0.9048	1.0000	0.9130
NPV	0.9733	0.8427	1.0000
FPR	0.0267	0.0000	0.0267
FNR	0.0952	0.6667	0.0000
FDR	0.0952	0.0000	0.0870
ACC	0.9583	0.8542	0.9792
F1	0.9048	0.5000	0.9545
MCC	0.8781	0.5300	0.9427
LRp	33.9300	Inf	37.5000
kappa	0.8781	0.4386	0.9411
TP	19.0000	7.0000	21.0000
TN	73.0000	75.0000	73.0000
FP	2.0000	0.0000	2.0000
FN	2.0000	14.0000	0.0000
counts	96.0000	96.0000	96.0000
